# Bile duct carcinoma associated with congenital biliary dilatation in a 16-year-old female: a case report and literature review

**DOI:** 10.1186/s40792-016-0132-y

**Published:** 2016-01-21

**Authors:** Hideki Izumi, Naoki Yazawa, Daisuke Furukawa, Yoshihito Masuoka, Misuzu Yamada, Taro Mashiko, Yohei Kawashima, Masami Ogawa, Yoshiaki Kawaguchi, Tetsuya Mine, Kenichi Hirabayashi, Toshio Nakagohri

**Affiliations:** Department of Gastrointestinal Surgery, Tokai University School of Medicine, 143 Shimokasuya, Isehara, Kanagawa 259-1193 Japan; Department of Internal Medicine, Tokai University School of Medicine, 143 Shimokasuya, Isehara, Kanagawa 259-1193 Japan; Department of Pathology, Tokai University School of Medicine, 143 Shimokasuya, Isehara, Kanagawa 259-1193 Japan

**Keywords:** Congenital biliary dilatation, Pancreaticobiliary maljunction, Bile duct carcinoma, Pancreaticoduodenectomy

## Abstract

We encountered a very rare case of bile duct carcinoma associated with congenital biliary dilatation (CBD) in a 16-year-old female who was admitted to our hospital because of right upper abdominal pain and vomiting. Abdominal computed tomography demonstrated a cystic dilatation of the common bile duct measuring 7 cm in diameter and two enhanced tumors 4 cm in diameter located in the inferior bile duct and middle bile duct. Magnetic resonance cholangiopancreatography clearly demonstrated a cystic dilatation of the extrahepatic bile duct (Todani’s CBD classification: type 4-A). Endoscopic retrograde cholangiopancreatography also revealed two tumors. Biopsy results of one of the tumors confirmed adenocarcinoma. Excision of the perihilar bile duct and subtotal stomach-preserving pancreaticoduodenectomy with dissection of the major lymph nodes were performed. A postoperative histopathologic examination revealed a well-differentiated tubular adenocarcinoma, which remained within the mucosal layer, and no lymph node metastasis was found. The postoperative course was uneventful, and the patient was discharged 10 days after surgery and has remained disease-free for 21 months.

## Background

It is well known that congenital biliary dilatation (CBD) has a significant association with bile duct carcinoma and that CBD merges with pancreaticobiliary maljunction (PBM) in all cases [1]. PBM is a congenital anomaly defined as a junction of the pancreatic and biliary ducts located outside of the duodenal wall that usually forms a markedly long common channel [2, 3]. Some authors have proposed a hyperplasia-dysplasia-carcinoma sequence [4, 5]. Thus, PBM is thought to be a high risk factor for the development of carcinoma in the biliary tract. Here, we report a rare case of a 16-year-old female with early bile duct carcinoma combined with PBM and CBD.

## Case presentation

A 16-year-old female was admitted to our hospital with a chief complaint of right upper abdominal pain and vomiting. Although our patient was autistic, her condition did not play any role in her present illness. Her body mass index was 18.49. Her family history included a grandfather treated for colon cancer at an unknown age, but there was no family history of anomalies of the pancreaticobiliary tract system. On physical examination, there was slight tenderness and an abnormal palatable mass in the right upper abdomen. Blood test findings on admission were as follows (Table 1): mild anemia and a longitude increase in hepatobiliary enzymes, amylase, and elastase 1, while levels of carcinoembryonic antigen and cancer antigen 19-9 (CA19-9) were within normal ranges.

**Table 1:** Blood test findings on admission

	Level	Units
WBC	4.9 × 10^3^	/μl
RBC	4.48 × 10^6^	/μl
Hb	10.5	g/dl
Ht	33.2	%
PLT	37.2 × 10^4^	/μl
BUN	12	mg/dl
Cr	0.59	mg/dl
Na	141	mEq/l
K	3.9	mEq/l
Cl	104	mEq/l
Ca	9.7	mg/dl
CRP	0.74	mg/dl
ALB	4.7	g/dl
CK	40	U/l
GOT	33	U/l
GPT	66	U/l
ALP	334	U/l
γ-GTP	103	U/l
T-Bil	0.9	mg/dl
D-Bil	0.3	mg/dl
AMY	138	U/l
CEA	1.2	ng/ml
CA19-9	29.0	U/ml
Elastase 1	437	ng/dl

**Fig. 1:**
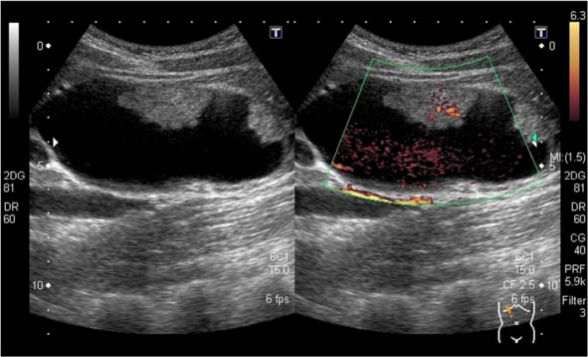
Abdominal ultrasonography showed two tumors extending into the common bile duct. Doppler ultrasound showed a blood flow signal within the tumor

**Fig. 2:**
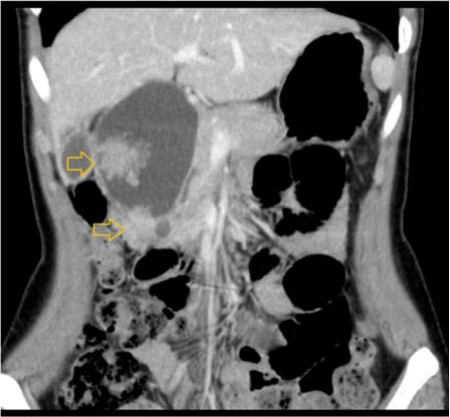
Abdominal CT showed two enhanced tumors (diameter, 4 cm) in the inferior and middle bile duct. *Yellow arrows* indicate the tumor locations

**Fig. 3:**
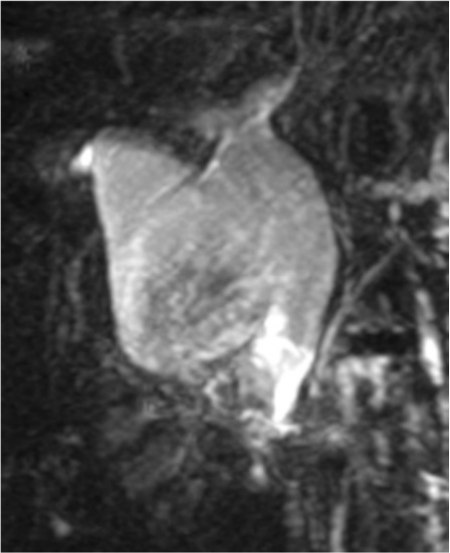
Magnetic resonance cholangiopancreatography demonstrated a cystic dilatation of the extrahepatic bile duct (Todani’s CBD classification: type 4-A)

**Fig. 4:**
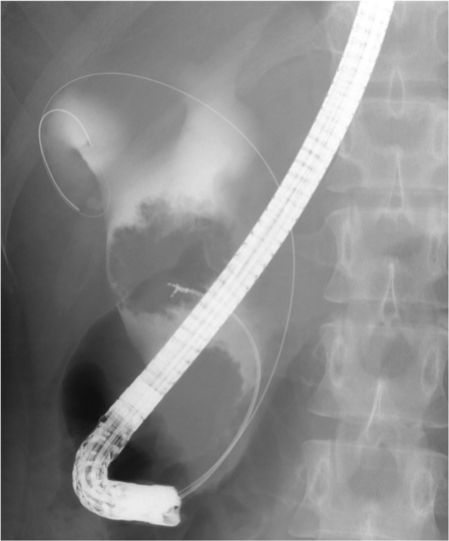
Endoscopic retrograde cholangiopancreatography showed two tumors, and biopsy of one of the tumors confirmed adenocarcinoma

Abdominal ultrasonography revealed a cystic dilatation of the extrahepatic bile duct and two protruding tumors in the lumen of the dilated common bile duct (Fig. 1). Doppler ultrasound detected a blood flow signal within the tumor. Abdominal computed tomography (Fig. 2) demonstrated a cyst within the common bile duct measuring 7 cm in diameter and two enhanced tumors measuring 4 cm in diameter located in the inferior bile duct and middle bile duct. Magnetic resonance cholangiopancreatography (Fig. 3) clearly demonstrated a cystic dilatation of the extrahepatic bile duct (Todani’s CBD classification: type 4-A) [1]. Endoscopic retrograde cholangiopancreatography (Fig. 4) also demonstrated a cystic dilatation of the bile duct as well as the presence of two tumors. Biopsy of one of the tumors confirmed the presence of adenocarcinoma. Amylase, CA19-9, and carcinoembryonic antigen levels in the choledochal cyst were 54,722 IU/l, 230,853 U/ml, and 3.051 ng/ml, respectively.

At laparotomy, the cystic common bile duct was easily found. The wall of the bile duct had no indurations or scaring. Excision of the perihilar bile duct and subtotal stomach-preserving pancreaticoduodenectomy (SSPPD) with dissection of the major lymph nodes were performed. The proximal stump of the choledochus was negative for malignant cells.

**Fig. 5:**
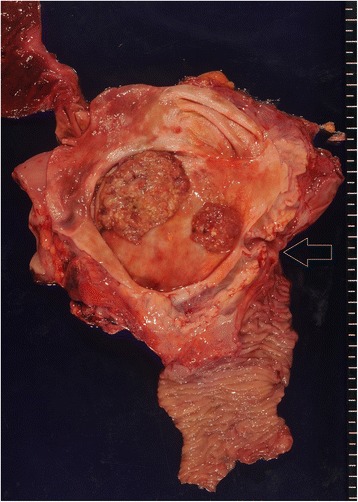
The resected specimen demonstrated tumors in the inferior and middle bile ducts. *Arrow* indicates the papilla of Vater

**Fig. 6:**
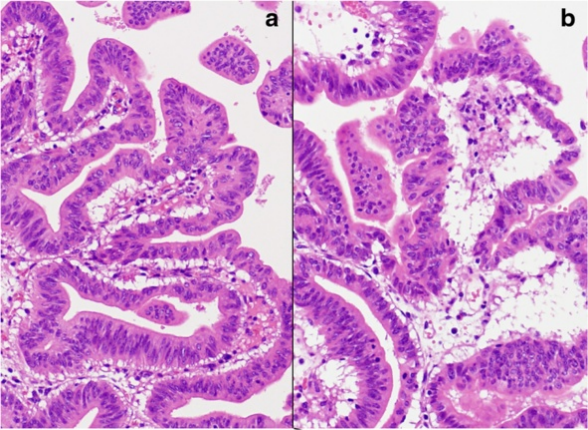
**a**, **b** Pathological examination revealed a well-differentiated tubular adenocarcinoma in the inferior bile duct (**a**) (hematoxylin and eosin (H.E., ×120) and middle bile duct (**b**) (H.E., ×120)

The resected specimen demonstrated tumors in the inferior and middle bile ducts (Fig. 5). Immunostaining was positive for CDX2 and negative for MUC1, MUC2, MUC5AC, MUC6, and p53. According to the Union for International Cancer Control (UICC), sixth edition rules, this case was stage 0 (Tis, N0, and M0). A postoperative histopathologic examination revealed a well-differentiated tubular adenocarcinoma, which entirely remained within the mucosal layer, and no lymph node metastasis was found (Fig. 6). The postoperative course was uneventful, and the patient was discharged 10 days after surgery and has remained disease-free for 21 months.

## Discussion

The disease concept of CBD and classification according to morphological features was first described by Alonso-Lej et al. in 1959 [6]. It is well known that CBD merges with PBM in all cases. PBM is a congenital anomaly defined as a junction of the pancreatic and biliary ducts located outside of the duodenal wall that usually forms a markedly long common channel [2, 3]. As the action of the sphincter of Oddi does not functionally affect this union, two-way regurgitation (biliopancreatic and pancreatobiliary reflux) occurs, resulting in various pathological conditions in the biliary tract and pancreas [7, 8]. PBM, particularly in Asian patients, is a well-known risk factor for the development of carcinoma in the biliary tract [9]. According to a report by Todani et al. [10], the mean patient age for carcinoma arising in a choledochal cyst is 37 years (range, 15–73 years). Therefore, advanced biliary tract carcinoma in teenage patients is relatively uncommon.

A MEDLINE literature search of English language articles revealed only six cases of biliary tract carcinoma in patients with PBM or CBD younger than 20 years (Table 2) [11–16]. Of these six patients, half were male and the most common PBM subtype was C-P, in which the common bile duct joins the major pancreatic duct. According to the classification of Todani [17], two patients had type 1a disease and two had type 4-A disease. The most common histological type of cancer was adenocarcinoma, and four cases underwent complete tumor resection of the choledochal cyst.

**Table 2:** Biliary tract carcinoma in patients with PBM or CBD younger than 20 years

No.	Author	Year	Sex	Age	Classification of Todani	Location of carcinoma	Operation	Histopathology	Outcome
1	Armanino	1946	Male	17	Unknown	Liver	Autopsy case	Adenocarcinoma	Dead
2	Fujiwara	1976	Female	17	Unknown	Choledochal duct cyst, mesenteric lymph node	Biopsy of a mesenteric lymph node	Adenocarcinoma	Dead
3	Iwai	1990	Female	12	4-A	Intrapancreatic duct	Cyst excision, hepaticojejunostomy	Adenocarcinoma	Dead
4	Tanaka	2006	Male	11	4-A	Cyst wall, common hepatic duct	Cyst excision, PpPD	Adenocarcinoma	Alive
5	Nakamura	2008	Female	15	1a	Cyst wall, liver metastases	PpPD, partial hepatectomy	Adenocarcinoma	Unknown
6	Saikusa	2009	Male	3	1a	Cyst wall	Cyst excision, hepaticojejunostomy	Adenocarcinoma	Alive
7	Our case	2015	Female	16	4-A	Cyst wall	Cyst excision, SSPPD	Adenocarcinoma	Alive

*PpPD* pylorus-preserving pancreaticoduodenectomy

In the present case, the bile amylase level in the common bile duct was 54,722 IU/l, so chronic inflammation leading to malignant change was observed. This high amylase level in the choledochal cyst suggested a free reflux of pancreatic juice into the biliary system that might have caused chronic inflammation in the choledochal cyst [18]. The carcinogenesis of CBD coexisting with PBM is considered to involve the hyperplasia-dysplasia-carcinoma sequence provoked by chronic inflammation resulting from a reflux of pancreatic juices into the biliary tract [4, 5]. Strong cytotoxic substances are produced when phospholipase A2 in the pancreatic juice mixes with bile, which have been recognized both clinically and experimentally to be injurious to cell membranes. Moreover, the subsequent chronic inflammation provokes high proliferative activity in the mucosal epithelia, finally resulting in CBD in PBM patients [19].

Once a diagnosis of CBD is made, early radical surgery for CBD combined with thorough intraoperative histopathologic examinations may be necessary, irrespective of the patient’s age. A preoperative diagnosis of bile duct carcinoma in CBD is sometimes difficult to establish [16]. In our case, the enhanced huge tumor in the dilated choledochus on CT was an important clue for the diagnosis of malignancy in CBD. The carcinoma spread to the level of the bile duct in the pancreas; therefore, after additional excision of the perihilar bile duct, SSPPD was performed. The surgical procedure should be modified based on the spread of the carcinoma because radical resection appears to be the only chance of cure in such cases.

## Conclusions

We encountered a case of bile duct carcinoma in a 16-year-old female with PBM and CBD. The incidence of cancer in young people with CBD is very low; however, CBD can become cancerous after a long period of time has elapsed. Therefore, at the time of diagnosis of CBD, a preoperative inspection for bile duct carcinoma may be necessary.

## Consent

Written informed consent was obtained from the patient’s parents for publication of this case report and all accompanying images. A copy of the written consent form is available for review from the Editor-in-Chief of this journal.

## Abbreviations

CA19-9: Cancer antigen 19-9
CBD: Congenital biliary dilatation
CT: Computed tomography
PBM: Pancreaticobiliary maljunction
SSPPD: Subtotal stomach-preserving pancreaticoduodenectomy
